# Evaluation of a super‐resolution deep learning reconstruction algorithm in abdominal CT imaging—A qualitative and quantitative performance analysis

**DOI:** 10.1002/acm2.70633

**Published:** 2026-05-26

**Authors:** Aria M. Salyapongse, Martin G. Wagner, Meghan G. Lubner, Kelsey L. Schluter, Matthew R. Smith, Michio D. Taya, Andrew L. Wentland, Timothy P. Szczykutowicz, Giuseppe V. Toia

**Affiliations:** ^1^ Departments of Medical Physics and Radiology University of Wisconsin–Madison Madison Wisconsin USA; ^2^ Department of Radiology University of Wisconsin–Madison Madison Wisconsin USA

**Keywords:** abdominal computed tomography deep learning image reconstruction, signal‐to‐noise, contrast‐to‐noise

## Abstract

**Background:**

A super‐resolution deep learning (DL) image reconstruction algorithm (Precise Image Quality Engine (PIQE)) was originally designed for cardiac CT, but is now available for abdominal CT.

**Purpose:**

To examine objective and subjective image quality (IQ) improvements PIQE compared to Advanced Intelligent Clear‐IQ Engine (AiCE) in abdominal CT.

**Methods:**

A retrospective analysis was conducted on 69 adult patient routine contrast enhanced abdominopelvic CT exams on a single Aquilion ONE/INSIGHT CT system (Canon Medical Systems, Otawara Japan). Images were reconstructed using PIQE (strength level L1 and L2) and AiCE (L1– the institutional standard). Four blinded radiologists assessed image noise, image contrast, small structure visibility, image sharpness, artifacts, and overall image preference with Likert scales. Reader agreement was assessed with Krippendorff's alpha. Circular regions of interest were placed on five slices on the left and right liver, portal vein, aorta, subcutaneous fat, and bilateral psoas muscles. CT number, noise, signal‐to‐noise ratios (SNRs), and contrast‐to‐noise ratios (CNRs) were determined. All significant differences between reconstructions were assessed via the Friedman test with post‐hoc Dunn‐Sidak corrections.

**Results:**

Reader agreement was fair ($\bar{\alpha }=0.20$). PIQE L2 was preferred for image contrast and image noise and PIQE L1 was preferred for image sharpness (*p* < 0.05). CT numbers were significantly different between AiCE L1 and PIQE (*p* < 0.05) and noise was statistically lowest in PIQE L2 compared to AiCE L1 (*p *< 0.05). SNR and CNR differences were statistically significant (*p* < 0.003), with PIQE L2 demonstrating the highest SNR and CNR.

**Conclusion:**

The best subjective IQ metrics for image contrast, image noise, and image sharpness were obtained with PIQE. The best objective IQ metrics (SNR and CNR) were obtained with PIQE L2. This work supports improved image contrast and decreased noise when using PIQE as compared to AiCE.

## 1 INTRODUCTION

Advances in artificial intelligence (AI) and deep learning (DL) have catalyzed a new era in computed tomography (CT) image reconstruction. Deep learning image reconstruction (DLR) attempts to overcome known limitations of traditional image reconstruction techniques like filtered back projection (FBP) and iterative reconstruction (IR).^1^, ^2^, ^3^ By using carefully curated training datasets, DLR has shown improvements over conventional techniques with respect to decreased noise magnitude, improved dose efficiency, lesion conspicuity, and maintained high contrast.^4^, ^5^ Both vendor‐specific (e.g., TrueFidelity [GE Healthcare, Waukesha, WI] and AiCE [Advanced intelligent Clear‐IQ Engine; Canon Medical Systems, Tustin, CA]) and vendor‐agnostic (e.g., PixelShine [AlgoMedica, Palo Alto, CA], ClariCT.AI [ClariPi, Rochester Hills, MI]) DLR CT reconstruction implementations are commercially available.

Prior DLR CT reconstruction work has primarily focused on improvements respective to FBP and IR.^4^, ^6^, ^7^, ^8^ Recently, a new DLR algorithm called Precise IQ Engine or PIQE (Canon Medical Systems, Tustin, CA) has become available.^9^, ^10^ PIQE not only advertises reduced image noise, but also synthetically enhanced spatial resolution. This DLR algorithm was trained using ultra‐high‐resolution data from the Aquilion Precision system (Canon Medical Systems) which utilizes 0.25 mm detector elements and a small (0.4 × 0.5 mm) focal spot, facilitating higher resolution data.^10^, ^11^, ^12^ PIQE training data included images reconstructed with AiCE (which was trained on model‐based iterative reconstruction images) in the super high resolution mode on the Aquilion Precision system (Canon Medical Systems, Tustin, CA).^10^ PIQE therefore allows image reconstruction with enhanced resolution on a non‐ultra‐high‐resolution system, specifically with a 1024 matrix.

To date, all literature assessing PIQE performance is in cardiac CT,^9^, ^11^, ^12^, ^13^ except work on dose reduction by Tamura et al. 2025.^14^ It is unknown how PIQE clinically performs in abdominopelvic CT (hereafter referred to as abdominal CT for simplicity). This work assesses PIQE image quality in abdominal CT using both subjective (radiologist reader analysis) and objective (signal‐to‐noise (SNR) and contrast‐to‐noise (CNR) ratios) assessments. To our knowledge, this is the first comprehensive evaluation of PIQE in abdominal CT using both quantitative and qualitative assessments.

## 2 METHODS

### 2.1 Study cohort and CT protocol

This was a Health Insurance Portability and Accountability Act compliant, retrospective study performed at a single quaternary academic medical center and approved by the University of Wisconsin School of Medicine and Public Health institutional review board. Informed consent was waived due to the retrospective nature of this work. The study cohort included 69 patients who had a contrast enhanced exam of the abdomen and pelvis performed between September to November 2024 and were 18 years or older. Bootstrapping ensured that this cohort size was large enough to detect the results. Study indications were varied and included indications across emergency, inpatient, and outpatient settings. Exclusion criteria were pediatric patients (less than 18 years of age) and any patient with metal implants that could potentially obscure anatomy of interest. Age, body mass index (BMI), weight, dose, and indication were recorded for the subjects.

Subjects were scanned with a routine contrast‐enhanced abdomen and pelvis protocols as detailed in Table 1 and based on the subject's anterior‐posterior (AP) and lateral (LAT) measurements as measured via localizer images. Intravenous iodinated contrast injections were performed using institutional weight‐based dose prescriptions. Subjects were injected with between 70–170 mL of Iohexol 350 mg I/mL (GEHealthCare, Waukesha, WI, USA) based on weight in pounds (130 to 320 lbs (59 to 145 kg), respectively) with increases of 5 mL for every 10 lbs (4.5 kg) greater than 130 lbs (59 kg). The injection protocol began with a saline test bolus of 3 mL/second, then the contrast volume, followed by a 50 mL saline flush at 3 mL/second. Circular regions of interest (ROI) were placed in the middle of the liver and monitored for the threshold value, and the scan was triggered when the ROI obtained a threshold of 200 Hounsfield units (HU) per institutional protocol.

**TABLE 1:** Scanner protocols for routine abdomen and pelvis.

Protocol	Patient size AP+LAT cm	kV	Pitch	Rotation time [s]	Automatic exposure control option
Small/Medium	<80	Sure kV^a^	0.806	0.75	Sure Exposure Control^b^ (60 to 590 mA)
Large	≥80		0.806	1.0	

^a^Vendor's automatic kV selection system.

^b^Vendor's automatic exposure control system. Standard deviation was set to 6.5 for the small/medium protocol and 7.5 for the large protocol.

Images were reconstructed using the institutional standard of AiCE strength level 1 (L1) with a 512 × 512 matrix and PIQE L1 and L2 with a 1024 × 1024 matrix. PIQE L3 was not included as this strength has only been recommended for CT angiography protocols. Slice thickness was 3 mm for both reconstruction methods. The AiCE algorithm, a deep convolution neural network, was trained using model‐based IR raw data from high dose scans in order to produce lower noise images from low dose scans.^2^, ^15^ The PIQE algorithm was trained using 0.25 mm ultra‐high resolution (UHR) images from the Aquilion Precision that were downsampled to yield images at the normal resolution (NR) of 0.5 mm. With both the UHR and NR images, the PIQE algorithm was trained to take in NR data and produce super high resolution (SHR) images with low noise.^16^, ^17^

### 2.2 Reader study

#### 2.2.1 Image analysis

In total, 207 image datasets (69 CT scans x 3 reconstructions) were anonymized such that no patient or scan information was available. Image analysis was performed on a dedicated research Picture Archiving and Communication (PACS) system (Change Healthcare; Nashville, TN).

#### 2.2.2 Qualitative analysis

Four board‐certified, fellowship trained abdominal radiologists (MGL, ALW, AW, MRS, MDT) with 16, 5, 2, and 2 years of post‐fellowship experience, respectively, performed qualitative image analysis in separate reading sessions with unlimited time. Readers were blinded to both study design and reconstruction identities and were asked to assess the image quality (IQ) according to the questions detailed in Table 2. Images were presented in a random order to prevent any potential biases. Images were assessed on high‐resolution calibrated monitors consistent with clinical standards and displayed at the default window settings (window width/level of 400/50). To simulate routine clinical workflow, readers were able to adjust window width/level settings based on personal preference and datasets were presented one‐by‐one.

**TABLE 2:** Likert scale questions for image quality analysis.

Assessment	Grade 1	Grade 2	Grade 3	Grade 4	Grade 5
Image noise	Unacceptable	Above average	Average	Less than average	Minimal
Image contrast	Very poor	Suboptimal	Acceptable	Above average	Excellent
Small structure visibility	Unacceptable	Suboptimal	Acceptable	Above average	Excellent
Image sharpness	Severe blurring, very poor edge definition, indiscernible margins	Moderate blurring, poor edge definition, discernible margins	Minimal blurring, good edge definition, easily discernible margins	No blurring, crisp edges, well‐defined margins	N/A^a^
Artifacts	Severe, affect diagnostic interpretation	Major but diagnostic interpretation possible	Minor, not affecting diagnostic interpretation	None	N/A^a^
Image preference	Would not interpret this image, unwilling to read in any clinical situation	Good for interpretation, willing to read in select clinical situations	Excellent for interpretation, willing to read in any clinical situation	N/A^‡^	N/A^‡^

^a^Indicates that this Likert grade was not used for this assessment.

Image noise, image contrast, and small structure visibility were assessed with a 5‐point Likert scale, image sharpness and artifacts were assessed with a 4‐point Likert scale, and image preference was assessed with a 3‐point Likert scale, using scales directly adapted from the European Guidelines on Quality Criteria for Computed Tomography.^18^ For all Likert scales, lower and higher values denoted worsening and improving IQ, respectively. Readers were provided with standardized instructions prior to the reading sessions and had the opportunity to ask questions or clarify the grading rubric (Table 2).

#### 2.2.3 Quantitative analysis

**FIGURE 1:**
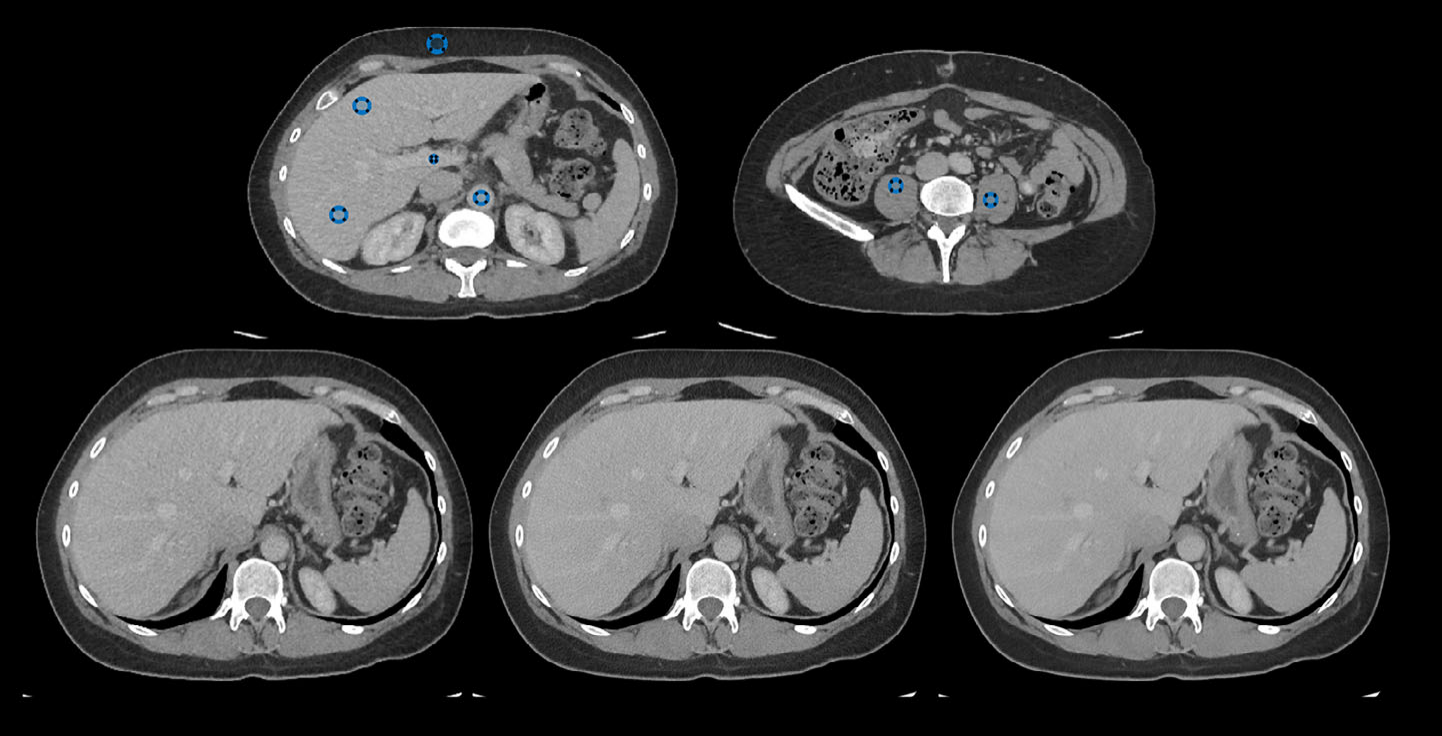
Top row: Example of region of interest placement. Bottom row: Example of axial images reconstructed with AiCE L1 (left), PIQE L1 (middle), and PIQE L2 (right). WW/WL of 400/40.

Circular ROIs were placed by a graduate student and a technologist (AMS, KLS) under the guidance of a radiologist not included in the reader study (GVT, 5 years’ post‐fellowship experience). An example of the ROI placements and example images of the three reconstruction algorithms are shown in Figure 1.

ROIs were placed in 5 sequential slices in each of the tissues of interest (left liver, right liver, portal vein, aorta, left psoas muscle, right psoas muscle) and subcutaneous fat to include as much tissue as possible while excluding extraneous structures (e.g., vessels in the liver and fat in the psoas muscles), edges, or artifacts. ROIs were propagated across image datasets to avoid potential variations in placement. Attenuation (in HU) was then recorded. A mean attenuation and standard deviation for each tissue type were calculated. SNR and CNR were calculated for each tissue of interest relative to the subcutaneous fat. Equations 1 and 2, respectively, define how SNR and CNR were calculated.

(1) $$SNR=\frac{CT_{\#,tissue}}{\sigma_{fat}}$$

(2) $$CNR=\frac{CT_{\#,tissue}-CT_{\#,fat}}{\sigma_{fat}}$$

where $CT_{\#,tissue}$ is the CT number of the tissue within the ROI (left liver, right liver, portal vein, aorta, bilateral psoas muscles), $CT_{\#,fat}$ is the CT number of the subcutaneous fat ROI, and $\sigma_{fat}$ is the noise (i.e., standard deviation of CT number) of the ROI.

### 2.3 Statistical analysis

Bootstrapping with 1001 random samples with replacement of the sample size (*n* = 69) was performed to ensure sufficient subject size for the statistical conclusions as described by.^19^ Trends between BMI or protocol and Likert score were evaluated with Spearman's rank correlation coefficient (BMI) or a Mann‐Whitney U test (protocol) to determine if BMI or protocol were potential confounding factors for analysis. Reader agreement was assessed using Krippendorff's alpha for multiple readers. Likert scores were tested for statistical differences using a Friedman test on the median scores from the four radiologist readers, with Dunn‐Sidak post hoc corrections for multiple comparisons. Statistical differences in CT number, noise, SNR, and CNR between the three reconstructions were assessed using the Friedman test with Dunn‐Sidak post‐hoc corrections for multiple comparisons. Interquartile ranges were recorded for Likert scores, CT numbers, noise magnitudes, SNR, and CNR. All statistical testing was performed in MATLAB (MATLAB version: R2023b), and a significance level of α = 0.05 was assumed for all analyses.

## 3 RESULTS

Sixty‐nine subjects comprised of 39 females and 30 males (mean age 54.5 ± 19.9 years; range 18–91 years) were included in the final patient cohort. Original scanning indications included: abdominal pain, evaluation for sepsis or small bowel obstruction, rectal pain, oncology evaluations, diverticulitis, flank pain, abdominal trauma, and postsurgical evaluations. The mean BMI was 31.0 ± 9.7 kg/m^2^ with range 18.3–60.4 kg/m^2^ (BMI was not available for five subjects). The mean weight was 85.9 ± 26.5 kg (range: 49.9–207.7 kg; weight was not available for one subject). Thirty‐eight subjects were scanned with the small/medium protocol and thirty‐one were scanned with the large protocol. The average CTDI_vol_ was 38.7 ± 11.1 mGy (dose length product: 2264.7 ± 727.0 cm*mGy; size‐specific dose estimate (SSDE): 40.5 ± 9.5 mGy). No statistically significant trend (|ρ|<0.2, *p *> 0.17) was found between Likert scores based on BMI, and no statistical difference was found between Likert scores based on protocol (*p* > 0.2). Additionally, since all subjects were compared to themselves between the reconstruction algorithms, heterogeneity in the dataset does not limit applicability. The following results are reported for median Likert scores between readers and average values are reported for CNR and SNR.

### 3.1 Qualitative analysis

Interreader agreement was fair on average ($\bar{\alpha }$ = 0.20), though overall scoring trends were similar among the four readers.

**FIGURE 2:**
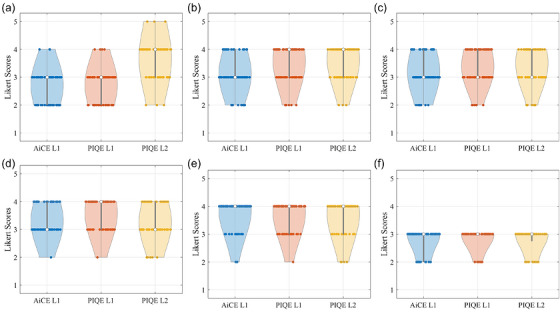
Violin plots of median Likert scores across four radiologist readers. (a) image noise, (b) image contrast, (c) small structure visibility, (d) image sharpness, (e) artifacts, (f) image preference.

Figure 2 demonstrates the median reader scores for each assessment over all three reconstructions. Statistical testing for median reader scores is summarized in Table 3. For image noise and image contrast, qualitative scores for PIQE L2 were statistically better compared to AiCE L1. For image contrast, PIQE L1 was preferred over AiCE L1. PIQE L2 scores were statistically better compared to PIQE L1 for image noise, but PIQE L1 was preferred over PIQE L2 for image sharpness. No statistical difference was observed in the Likert scores for small structure visibility, artifacts, or image preference for any of the comparisons. Figure 2 and Table 3 corroborate with respect to PIQE L2 preferred for image noise, PIQE L1 and L2 preferred for image contrast, and PIQE L1 preferred for image sharpness. In no cases was AiCE L1 preferred over PIQE L2.

**TABLE 3:** Likert score assessments across tested DLR algorithms. Median and interquartile ranges (IQR) are reported for the listed reconstructions.

	AiCE L1 v. PIQE L1		AiCE L1 v. PIQE L2		PIQE L1 v. PIQE L2	
Assessment	AiCE L1 Median [IQR]	*p*	PIQE L2 Median [IQR]	*p*	PIQE L1 Median [IQR]	*p*
Image noise	3 [1]	0.98	4 [1]	**A**	3 [1]	**A**
Image contrast	3 [1]	**0.02**	4 [1]	**0.0002**	4 [1]	0.44
Small structure visibility	3 [1]	0.48	4 [1]	0.07	3 [1]	0.71
Image sharpness	3 [1]	0.06	3 [1]	0.95	4 [1]	**0.02** *^⁑^*
Artifacts	4 [0]	0.98	4 [1]	0.33	4 [1]	0.55
Image preference	3 [1]	0.34	3 [0]	0.96	3 [0]	0.63

*Note*: Friedman test on the median scores with Dunn‐Sidak post hoc corrections for multiple comparisons was used.

**A** indicates that the *p‐*value was <0.00005

**Bold face** indicates statistical significance (*p* < 0.05) of second listed algorithm over first listed algorithm.

**Bold face** with ⁑ indicates PIQE L1 was preferred over PIQE L2 for this assessment.

Bootstrapping indicated that a sample size of 69 provided greater than 95% power to detect significant differences in image noise, 66–95% power to detect significant differences in image contrast, and 9% power to detect significant differences in image sharpness between the three reconstruction algorithms assuming a statistical significance level of 5%.

### 3.2 Quantitative analysis

**FIGURE 3:**
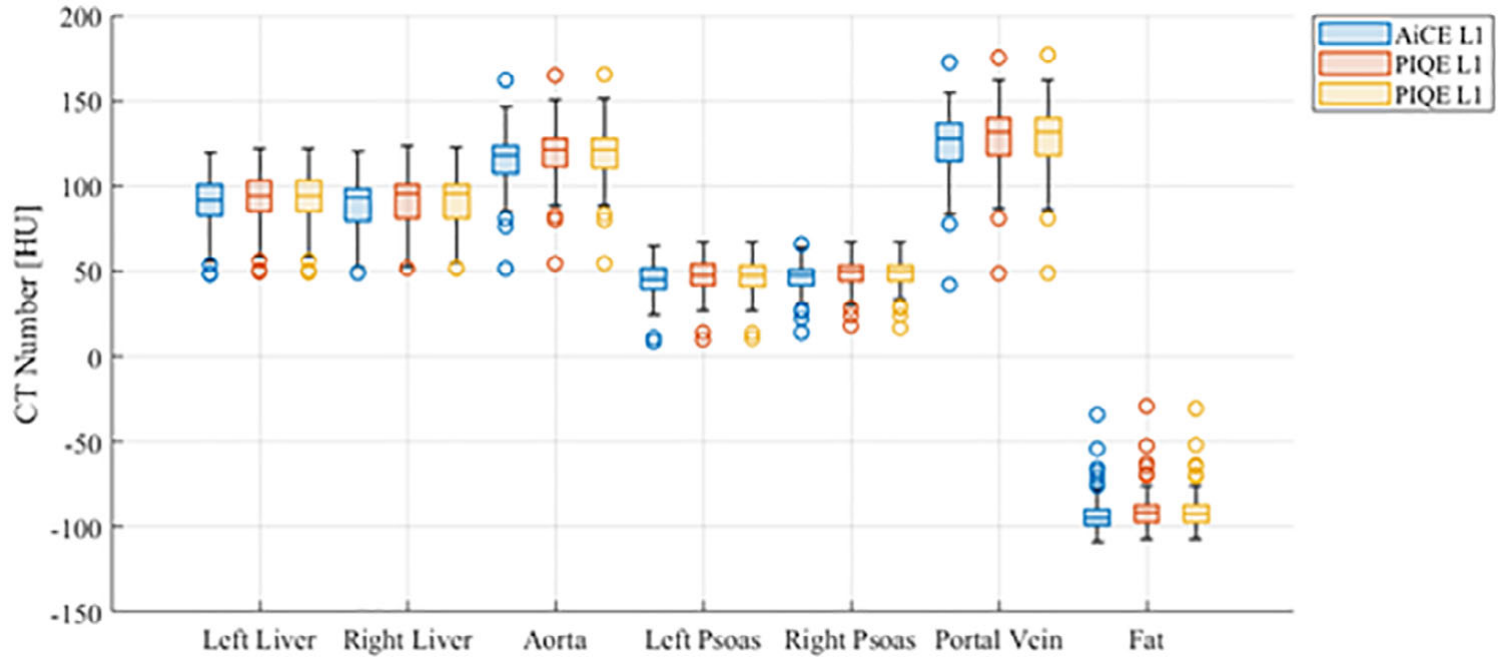
Box plots of mean CT number (in Hounsfield units (HU)) for left liver, right liver, aorta, left psoas, right psoas, portal vein, and fat regions of interest with AiCE L1 (blue), PIQE L1 (red), and PIQE L2 (yellow) reconstructions.

**Figure** **3** demonstrates the CT numbers of the ROI locations and different reconstruction algorithms. Table 4 lists the results of comparing CT numbers and noise of the DLR reconstructions. CT numbers were statistically difference between AiCE L1 and PIQE (L1 and L2) for all tissues, but on average the difference between the reconstructions was less than 4 HU, with PIQE reconstructions having higher CT numbers. Noise was statistically different among the three DLR algorithms, with PIQE demonstrating less noise than AiCE in all tissues (*p *< 0.05), except the left psoas muscle, portal vein, and fat for AiCE L1 compared to PIQE L1.

**TABLE 4:** CT number and noise mean values across tested DLR algorithms. Median and interquartile ranges (IQR) are reported for the listed reconstructions.

Tissue	AiCE L1 v. PIQE L1				AiCE L1 v. PIQE L2				PIQE L1 v. PIQE L2			
	AiCE L1 CT number		AiCE L1 Noise		PIQE L2 CT number		PIQE L2 Noise		PIQE L1 CT number		PIQE L1 Noise	
	Mean [IQR]	*p*	Mean [IQR]	*p*	Mean [IQR]	*p*	Mean [IQR]	*p*	Mean [IQR]	*p*	Mean [IQR]	*p*
Left liver	90.7 [18.2]	**A**	2.1 [1.0]	0.05^•^	93.2 [18.5]	**A**	2.0 [1.2]	**A** ^•^	93.2 [18.2]	0.14	2.0 [0.9]	0.10
Right liver	88.9 [19.5]	**A**	2.0 [1.2]	**0.01^•^**	91.3 [19.6]	**A**	1.8 [1.0]	**A** ^•^	91.5 [19.5]	**0.04** ^•^	1.8 [1.1]	**0.0003^•^**
Aorta	114.4 [15.7]	**A**	2.7 [1.3]	**0.0002** ^•^	118.0 [16.6]	**A**	2.2 [1.5]	**A** ^•^	118.0 [16.7]	0.49	2.2 [1.4]	**0.02** ^•^
Left psoas	44.5 [12.0]	**A**	2.5 [1.8]	0.29	47.0 [12.5]	**A**	2.4 [1.7]	**0.004** ^•^	47.0 [12.3]	0.91	2.4 [1.6]	0.28
Right psoas	46.2 [9.4]	**A**	2.8 [1.3]	**0.0008** ^•^	48.7 [9.7]	**A**	2.6 [1.4]	**A** ^•^	48.2 [9.5]	0.99	2.6 [1.3]	0.61
Portal vein	123.9 [22.1]	**A**	4.2 [2.6]	0.21	127.8 [22.2]	**A**	3.6 [2.3]	**A** ^•^	127.6 [22.4]	0.10	4.0 [2.9]	**A** ^•^
Fat	−91.9 [9.0]	**A**	3.6 [2.4]	0.99	−89.8 [9.8]	**A**	3.5 [2.2]	**0.0004** ^•^	−90.1 [9.7]	**A**	3.4 [2.4]	**0.001** ^•^

*Note*: Friedman test on the mean values with Dunn‐Sidak post hoc corrections for multiple comparisons was used.

**A** indicates that the *p‐*value was <0.00005

**Bold face** indicates a statistically significant difference (*p* < 0.05) between the two algorithms.

**Bold face** with ^•^ indicates a statistically significant difference (*p* < 0.05) between the two algorithms, with the first listed algorithm have a higher value (i.e., higher CT number or higher noise) than the second listed algorithm.

**FIGURE 4:**
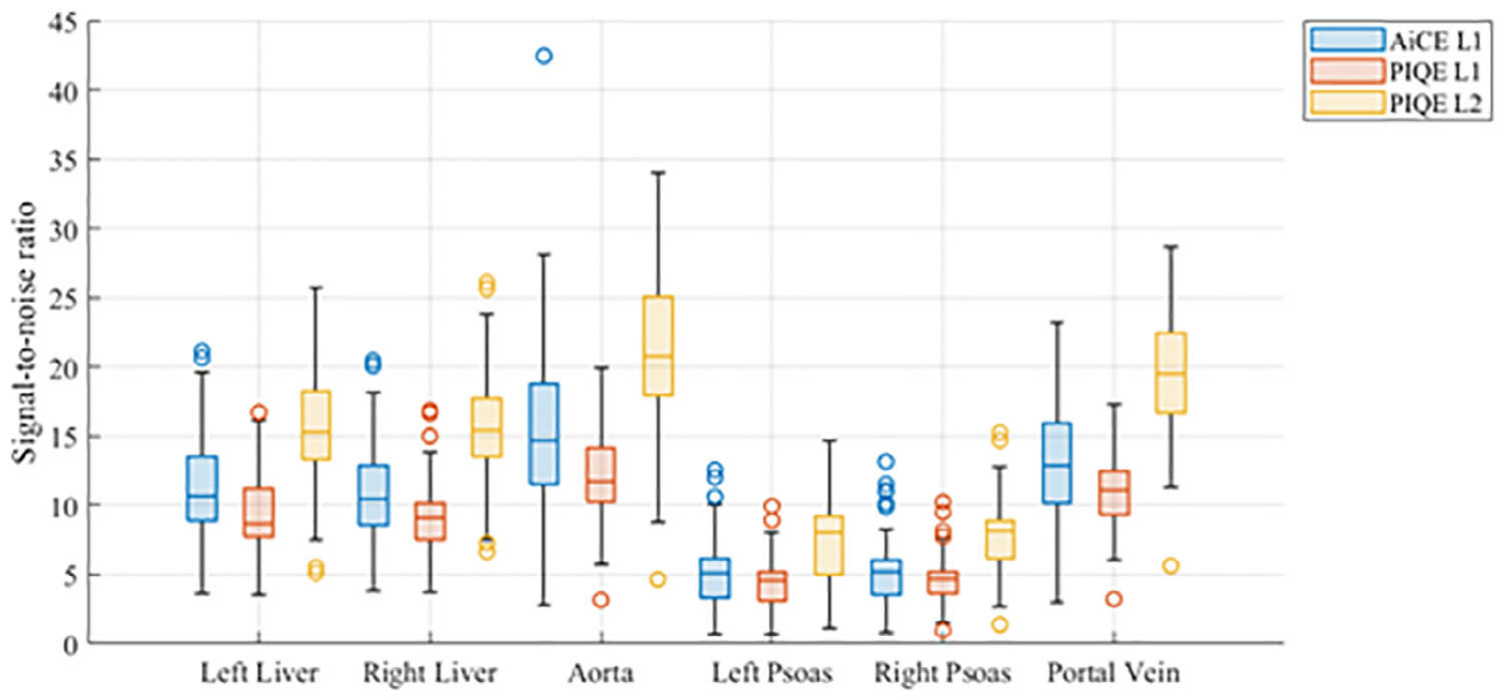
Box plots of mean signal‐to‐noise ratio (SNR) for left liver, right liver, aorta, left psoas, right psoas, and portal vein regions of interest with AiCE L1 (blue), PIQE L1 (red), and PIQE L2 (yellow) reconstructions.

**FIGURE 5:**
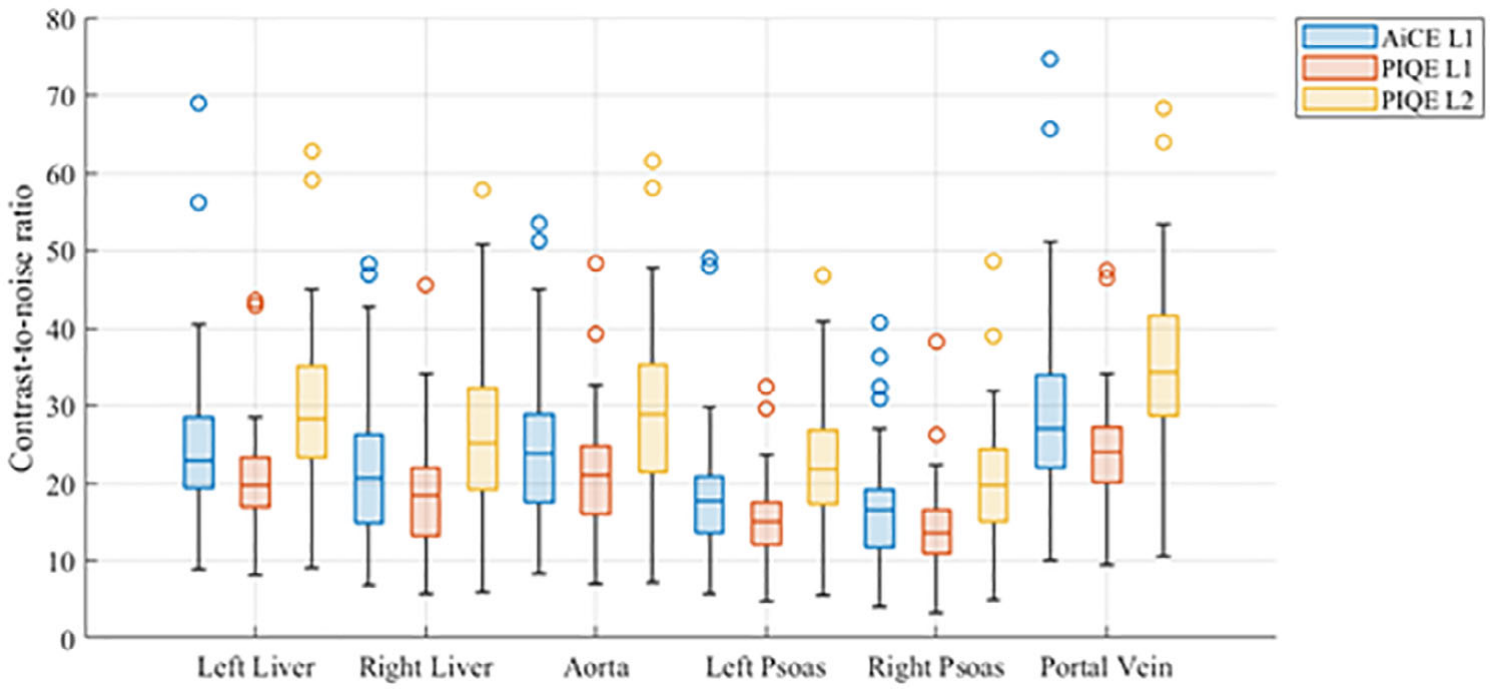
Box plots of mean contrast‐to‐noise ratio (CNR) for left liver, right liver, aorta, left psoas, right psoas, and portal vein regions of interest with AiCE L1 (blue), PIQE L1 (red), and PIQE L2 (yellow) reconstructions.

SNR and CNR for each assessed tissue are demonstrated in Figures 4 and 5, respectively. Table 5 lists the results of statistical testing used to determine if there was a statistically significant difference between SNR and CNR of any of the DLR reconstructions.

**TABLE 5:** Signal‐to‐noise (SNR) and contrast‐to‐noise (CNR) values between DLR reconstructions. The listed p‐values are for tests that demonstrate superiority of the second listed reconstruction over the first listed reconstruction unless otherwise specified.

	AiCE L1 v. PIQE L1				AiCE L1 v. PIQE L2				PIQE L1 v. PIQE L2			
	AiCE L1 SNR		AiCE L1 CNR		PIQE L2 SNR		PIQE L2 CNR		PIQE L1 SNR		PIQE L1 CNR	
Tissue	Mean [IQR]	*p*	Mean [IQR]	*p*	Mean [IQR]	*p*	Mean [IQR]	*p*	Mean [IQR]	*p*	Mean [IQR]	*p*
Left liver	11.5 [4.6]	**A** ^⁂^	24.5 [9.3]	**A** ^⁂^	15.9 [4.9]	**A**	29.5 [12.0]	**A**	9.5 [3.5]	**A**	20.4 [6.6]	**A**
Right liver	11.0 [4.3]	**A** ^⁂^	21.5 [11.4]	**A** ^⁂^	15.5 [4.2]	**A**	25.9 [13.1]	**A**	9.2 [2.6]	**A**	18.2 [8.9]	**A**
Aorta	15.5 [7.2]	**A** ^⁂^	24.4 [11.7]	**A** ^⁂^	21.3 [7.2]	**A**	29.7 [13.9]	**A**	12.1 [3.9]	**A**	20.8 [9.0]	**A**
Left psoas	5.1 [2.8]	**0.003** ^⁂^	18.4 [7.3]	**A** ^⁂^	7.4 [4.2]	**A**	22.2 [9.5]	**A**	4.4 [2.0]	**A**	15.3 [5.3]	**A**
Right psoas	5.3 [2.5]	**0.001**	16.4 [7.6]	**A** ^⁂^	4.7 [2.7]	**A**	19.9 [9.3]	**A**	7.8 [1.6]	**A**	14.0 [5.6]	**A**
Portal vein	13.2 [5.8]	**A** ^⁂^	28.8 [11.9]	**A** ^⁂^	19.5 [5.8]	**A**	34.9 [13.1]	**A**	11.0 [3.2]	**A**	24.1 [7.2]	**A**

*Note*: Friedman test on the mean values with Dunn‐Sidak post hoc corrections for multiple comparisons was used.

**A** indicates that the *p‐*value was <0.00005

**Bold face** indicates statistical significance (*p* < 0.05) of second listed algorithm over the first listed algorithm.

**Bold face** with ^⁂^ indicates statistical significance (*p* < 0.05) of first listed algorithm over the second listed algorithm.

Bootstrapping indicated that a sample size of 69 provided greater than 95% power to detect significant differences in SNR and CNR.

All comparisons demonstrated that AiCE L1 has higher SNR and CNR than PIQE L1 (*p *< 0.003). All comparisons demonstrated that PIQE L2 had higher SNR and CNR compared to AiCE L1 (*p *< 0.00005), and PIQE L1 (*p *< 0.00005).

## 4 DISCUSSION

This work demonstrated that a new DLR algorithm (PIQE) was preferred over the existing vendor‐specific standard of care (AiCE) in terms of image contrast, image noise, and images sharpness across radiologist readers. PIQE L2 had superior SNR and CNR over PIQE L1, but PIQE L1 was subjectively preferred in terms of image sharpness. While a difference in CT number between the reconstruction techniques was observed, this change in CT number is statistically significant but may not be clinically significant.

DLR algorithms like AiCE have several demonstrable advantages in abdominal and pelvic imaging.^4^, ^20^ These advantages include reduced noise ^11^, ^21^, ^22^, ^23^, ^24^ suppression of artifacts,^25^ preservation of FBP‐like noise texture,^7^ and higher dose efficiency ^11^, ^21^, ^23^, ^26^ without slow reconstruction times.^20^ However, AiCE still struggles with some tasks such as preservation of small lesions at low doses, where Zhang et al. 2022 found that AiCE reconstructions missed some small (< 3 mm) renal calculi.^27^ Therefore, new DL algorithms could be useful for the tasks that current algorithms struggle with, including potential improvements in bariatric imaging.^24^ The PIQE algorithm has been specifically trained to produce high resolution images with low noise.^16^, ^17^ Therefore, use of this algorithm was expected to demonstrate improvements in spatial resolution and noise compared to AiCE, which was observed in the improved scores for image sharpness and image noise, respectively, in the radiologist scores.

Most work assessing PIQE image quality derives from cardiac applications^9^, ^12^, ^13^ due to the need for higher resolution in cardiac tasks, however some groups have demonstrated similar analyses in abdominal CT with PIQE despite abdominal high‐resolution tasks not being as well‐defined.^14^ Tamura et al. 2025 examined noise magnitude, CNR, noise‐power spectrum (NPS), edge rise slope, and subjective evaluation by radiologists with low dose protocols in both hybrid iterative reconstruction (HIR) and PIQE.^14^ Similar to the results presented in this work, this group found that AiCE L1 demonstrated improved CNR compared to PIQE L1 but lacked assessment of PIQE L2 compared to AiCE. In this work, PIQE L2 demonstrated the best SNR and CNR compared to AiCE L1 and PIQE L1. This group also demonstrated that radiologists preferred PIQE for image noise, artifacts, sharpness, and image preference with statistical significance, which contrasts with the results of the radiologist scoring in this work, where PIQE L1 and L2 were preferred for noise and contrast, but not for artifacts, sharpness, or image preference. Additionally, this group suggested that PIQE could be useful for detection of small, low‐contrast lesions which can occasionally be affected by noise texture shifts away from filtered back projection‐like noise texture.^14^, ^28^

Other groups have performed similar analyses of PIQE in cardiac imaging.^11^, ^12^, ^13^ Nagayama et al. 2023 analyzed the CNR of AiCE and PIQE in the left main trunk, right coronary artery, left anterior descending artery, and left circumflex artery and asked radiologists to assess image sharpness, noise, and overall quality.^11^ This group found that PIQE demonstrated higher CNR and higher subjective radiologist evaluations, similar to what was demonstrated in this abdominal PIQE work. The same group published another work in the American Journal of Roentgenology ^12^ that again demonstrated improved CNR and subjective IQ metrics for the PIQE reconstructions compared to the AiCE reconstructions. Finally, Orii et al. 2023 demonstrated that PIQE provided higher SNR and CNR in coronary CT angiography compared to a model‐based IR algorithm from the same vendor (FIRST, Canon Medical Systems).^13^

Further improvements in SNR and CNR, including decreased noise, can improve the clinical utility of exams, such as improving lesion detection, reducing dose, and aiding in bariatric imaging.^3^, ^4^, ^7^ Therefore, use of PIQE could affect protocol parameters for specific applications in CT.

There are several limitations for this study. First, this work was performed only with one site, one patient population, and two scanning protocols with only axial images assessed, therefore these results may not be generalizable. Second, while the reader agreement was poor as assessed by Krippendorff's alpha, there were similar trends of high scores for PIQE L2 on image noise and contrast among the four readers. Third, this study does not include a full physics‐based analysis of the system and reconstruction options, which could help elucidate some of the utility or limitations of the reconstruction algorithm. Fourth, this study did not evaluate the effect of matrix size on the performance of the algorithm and only assessed the standard matrix sizes for AiCE and PIQE, which are 512 and 1024, respectively. Since matrix size could be a confounding variable for noise and spatial resolution, this limits the generalizability of this work. Specifically, smaller pixels would be more susceptible to noise and represent better spatial resolution compared to larger pixels. However, the 1024 matrix size was only available with PIQE and the goal of this work was to compare PIQE 1024 to current reconstruction algorithms, which are limited to a 512 matrix. Finally, in this work we did not perform an objective evaluation of spatial resolution to assess the synthetic enhanced spatial resolution, though this work is currently underway. Future work also includes a full physics analysis and evaluation of potential dose‐escalation in abdominal CT with PIQE as discussed by other groups and an assessment of diagnostic accuracy with the three reconstructions.^14^

## 5 CONCLUSION

This work showed that PIQE L2 reconstruction algorithm demonstrated improved SNR and CNR compared to PIQE L1 and to the institutional standard of AiCE L1. PIQE L2 was also rated with improved image contrast and lower noise, aligning with the claimed benefits of this new DL reconstruction algorithm. This works overall supports that PIQE further improves upon the existing benefits of AiCE regarding noise, CNR, and SNR. However further quantitative testing regarding spatial resolution claims and evaluation of diagnostic accuracy should be performed.

## AUTHOR CONTRIBUTIONS

Methodology and data collection was performed by Aria M. Salyapongse, Kelsey L. Schluter, and Giuseppe V. Toia. Data analysis was performed by Aria M. Salyapongse, Martin G. Wagner, Meghan G. Lubner, Matthew R. Smith, Timothy P. Szczykutowicz, Michio D. Taya, Andrew L. Wentland, and Giuseppe V. Toia. All authors contributed to writing and editing the manuscript.

## CONFLICT OF INTEREST STATEMENT

Aria M. Salyapongse, Kelsey L. Schluter, Matthew R. Smith, Andrew L. Wentland, and Michio D. Taya declare no conflict of interest. Martin G. Wagner has financial relationships with HistoSonics Inc. (consultant, research support), Siemens Healthineers (research support), and Canon Medical (research support). Meghan G. Lubner spouse consultant, Elphas Bio. Giuseppe V. Toia is a consultant for Canon Medical Systems. Timothy P. Szczykutowicz receives research support from Canon Medical Systems and GE HealthCare, consulting fees from Alara Imaging, Imalogix; royalties from Medical Physics Publishing and royalties related to intellectual property from Qaelum; founder of RadUnity.

## FUNDING INFORMATION

The authors did not receive support from any organization for the submitted work.

## ETHICS STATEMENT

The University of Wisconsin—Madison Institutional Review Board waived consent due to the retrospective nature of this work.

## Data Availability

All data are included in the manuscript.
